# Study of the Forming Characteristics of Small-Caliber Ammunition with Circumferential MEFP

**DOI:** 10.3390/ma13040891

**Published:** 2020-02-17

**Authors:** Guangsong Ma, Guanglin He, Yukuan Liu, Yachao Guo

**Affiliations:** School of Mechatronical Engineering, Beijing Institute of Technology, Beijing 10081, China; kfmaguangsong@163.com (G.M.); yukuanliu@foxmail.com (Y.L.); 3120160128@bit.edu.cn (Y.G.)

**Keywords:** Small-caliber ammunition, integral circumferential MEFP, variable wall thickness, numerical simulation, forming characteristics

## Abstract

To study the influence of the structural parameters of the ammunition liner of small-caliber ammunition on the forming characteristics of the projectile, an integrated circumferential multiple explosively formed projectile (MEFP) warhead with an integrated shell and the liner was initially designed, and the wall thickness of the liner is variable. LS-DYNA finite-element software is used to simulate the integral circumferential MEFP of the preliminary design, based on the numerical simulation results, the influence of the thickness at the center of the liner, and the curvature radius of the liner on the shape and velocity of the formed projectile. The numerical simulation results show that when the thickness of the center of the liner is constant and the curvature radius increases gradually, the velocity of the formed projectile decreases and the length: Diameter ratio of formed projectile decreases gradually. When the curvature radius of the liner remains unchanged, the velocity of the formed projectile decreases with the increase of the thickness of the center of the liner, and the shape of the formed projectile does not change significantly. The results show that when the design of integrating the shell and the liner was adopted for the integral circumferential MEFP warhead, the shape of the formed projectile is greatly affected by the curvature radius of the liner (curvature radius of inner and outer walls of the liner), but less by the thickness of the center of the liner. The velocity of the formed projectile is affected by the curvature radius of the inner and outer walls of the liner and the thickness of the center of the liner. Moreover, the influence of the thickness of the center of the liner on the velocity of the formed projectile is greater than that of the curvature radius of the outer wall of the liner.

## 1. Introduction

Conventional small-caliber ammunition is used to destroy the target with fragments generated during the explosion. However, in the face of armed personnel with stronger protective performance, fragments alone cannot cause enough damage to them. To penetrate the armed personnel with better protection, the fragments formed by small-caliber grenades need more kinetic energy, and there are two ways to improve the kinetic energy, i.e., to improve the mass of the fragments, and increase the speed of the fragments. For small-caliber ammunition, the detonation energy generated by the charge is also fixed due to the size and the fixed charge quantity. If the mass of the fragment is increased, the fragment flying velocity will inevitably decrease; if the mass of fragments is reduced, the speed of fragments can be improved to some extent, but the improvement of the ability of armed personnel with stronger penetration protection capability is still limited. A large number of small-caliber ammunition fragments and, despite many fragments hitting the target, the kinetic energy of each fragment is too small to penetrate the protective equipment of the armed personnel. Since the 1980s, researchers from all over the world have studied multiple explosively formed projectile (MEFP) warheads [1]. Therefore, the small-caliber ammunition has been designed as the structure of MEFP. Although the number of the final formed projectile is limited, the projectile velocity is very high. With the improvement of the precision of the proximity fuse, the probability of a limited number of formed projectiles hitting the armed personnel is also increased, so that the projectiles can not only hit the armed personnel, but also penetrate the protective equipment of the armed personnel. At present, the research on MEFP warheads can be divided into two categories in the dispersion direction of formed projectiles. The first one is that the liner is arranged along the axis of the charge [2,3,4,5,6,7,8,9], and the explosively formed projectile (EFP) flies away in the axial direction of the charge; the second one is that the liner is arranged along the circumferential direction of the charge [10,11,12,13,14], and the EFP flies along the radial direction of charge. In related literature [2,3,4,5,6,7,8,9,15,16,17], there are many research works on the forming characteristics of the projectile when it is scattered along the charge axis. Li Peng [13] et al. applied eccentric initiation technology to the MEFP warhead through static detonation experiment and numerical simulation, and found that the eccentric initiation could improve the molding results of MEFP, and greatly improved the velocity of the shaped projectile and the penetration depth of the target plate in the directional region. Liang Zhen Gang et al. [11,12] studied the forming law of MEFP from the aspect of parameters of the liner by simplifying the model of the MEFP warhead and combining numerical simulation with experiment, and found that the thickness of the liner affected the forming velocity of the projectile, and the thickness of the liner and the radius of curvature together affected the shape of the projectile. Yin Jianping [13] et al. analyzed the forming law of the MEFP warhead from the aspect of the parameters of the liner by numerical simulation, the influence of the parameters of the liner on the formation of the circumferential MEFP was studied, by statistical analysis of the formed assembly EFP, the regulation of the curvature radius, thickness, and caliber of the liner’s influence to the forming of circumferential MEFP, and the appropriate relatives among parameters were obtained. Zheng Can Jie [14] et al. studied the arrangement, number of layers, and initiation mode of the liner by numerical simulation. The results show that the comprehensive performance of staggered sub-EFP is better than that of parallel sub-EFP; the velocity of sub-EFP increases with the increase of the number of layers arranged in the liner; the axial velocity of sub-EFP formed by line initiation is higher than that of three-point initiation.

In the study of the MEFP warhead in the literature [2,3,4,5,6,7,8,9,10,11,12,13,14,15,16,17], there are two significant features. One is that the wall thickness is equal to the shell and liner, and the other is that the liner is designed separately from the shell. For the small-caliber ammunition with large demand, because the size of the liner is very small, if the shell and liner separated design is adopted, the production efficiency may be affected in mass production. However, if the shell and liner integrated design is adopted, the production process will be simplified, and the production efficiency will be improved. In the study of MEFP warhead forming characteristics in the literature [2,3,4,5,6,7,8,9,10,11,12,13,14,15,16,17], the wall thickness is equal to the shell and liner, and the forming characteristics of liner with variable wall thickness are rarely reported. Therefore, LS-DYNA software will be used in this study to simulate the forming characteristics of the circumferential MEFP when the liner is of variable wall thickness.

## 2. Structural Design and Analysis

**Figure 1:**
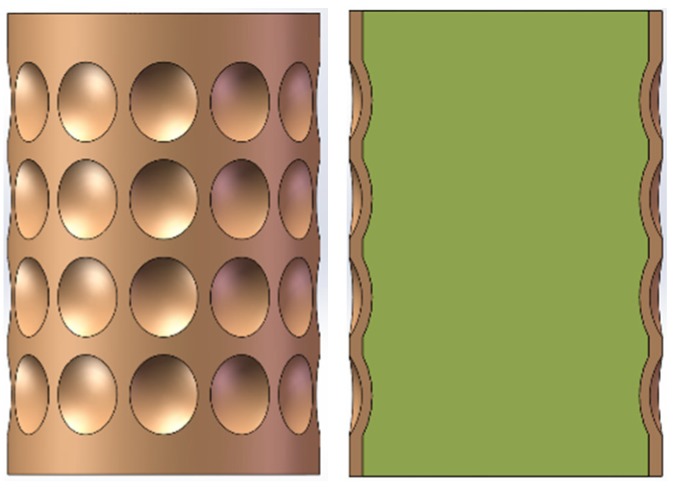
Structural design of integral circumferential MEFP warhead.

In the study of the molding characteristics of the circumferential MEFP in the literature [13,14,15,16,17], the influence of the change of the relevant parameters of the liner on the forming characteristics of the MEFP is mainly analyzed. Therefore, in this study, the design of the integral circumferential MEFP warhead is shown in Figure 1 when the liner has a certain caliber. The charging height is 52 mm, the diameter of the charging is 37 mm, the liners are arranged along the charging circumference, 4 rows in total along the charging axis, 12 liners in each row along the charging radial direction, giving a total of 48 liners.

To study the forming characteristics of the projectile when the liner is of variable wall thickness, the parameters of the shell and liner are designed as shown in Table 1. When the curvature radius of the outer wall of the liner is less than the curvature radius of the inner wall, the thickest part of the liner is less than the shell thickness. The wall thickness of the shell part is 1.5 mm, the diameter of the liner is 9 mm, and the thickness of the center of the liner is 1 mm, 1.15 mm and 1.3 mm respectively; the curvature radius of the inner wall of the liner is 8 mm, 10 mm, 12 mm and 14 mm respectively, and the curvature radius of the outer wall of the liner is 6 mm, 8 mm, 8.5 mm and 12 mm respectively, as shown in Table 1.

**Figure 2:**
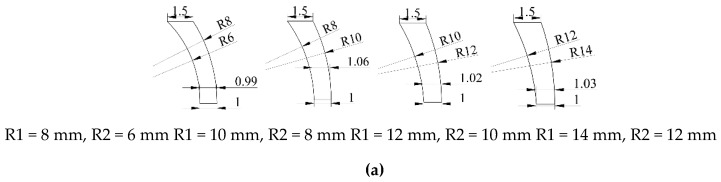

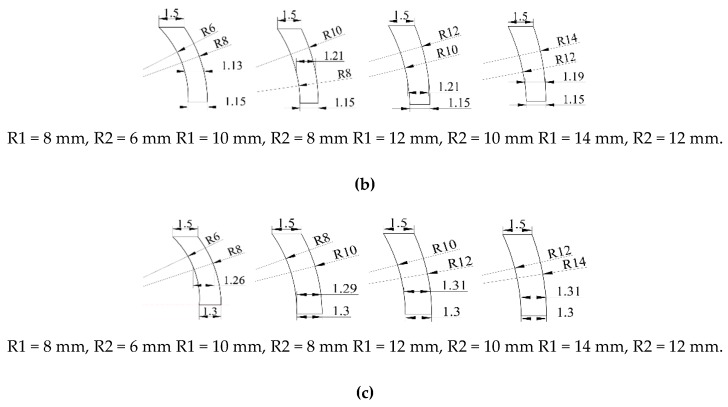
Structure diagram of the liner: (**a**) Structural diagram of the liner with a thickness of 1 mm at the center; (**b**) Structural diagram of the liner with a thickness of 1.15 mm at the center; (**c**) Structural diagram of the liner with a thickness of 1.15 mm at the center.

The structure diagram of the liner of the circumferential MEFP charge shown in Table 1 is shown in Figure 2.

It can be seen from the structural diagram shown in Figure 2 that the shell thickness of the integral MEFP ammunition designed by the integration of shell and liner is 1.5 mm, while the thickness at the center are 1 mm, 1.15 mm and 1.3 mm respectively. Therefore, when the curvature center of inner wall of liner is in the same straight line with the center of liner, the curvature center of outer wall of liner deviates from the straight line. Therefore, when R1 = 8 mm, R2 = 6 mm, with the increase of the thickness at the center of the liner, the thickness from the center to the edge of the liner first decreases and then increases, and the thinnest part of the liner is gradually away from the center of the liner. When R1 = 10 mm, R2 = 8 mm, and Δ t = 1.3 mm, the thickness of the liner first decreases and then increases. When R1 = 10 mm, R2 = 8 mm, Δ t = 1 mm, 1.15 mm, the thickness of the liner increases gradually from the center to the edge. When R1 = 12 mm, 14 mm respectively, R2 = 10 mm, 12 mm respectively, with the increase of the center thickness of the liner, the liner thickness gradually increases from the center to the edge.

## 3. Numerical Model of Integral Circumferential MEFP Warhead

### 3.1. Finite-Element Model of Integral Circumferential MEFP Warhead

**Figure 3:**
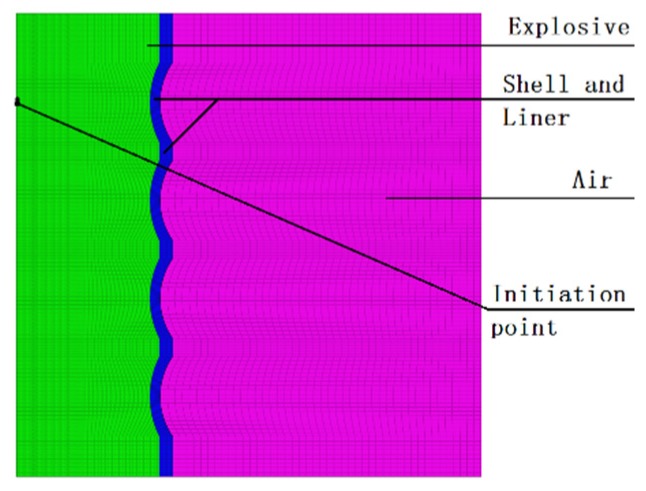
Finite-element model of integral circumferential MEFP warhead.

At present, the mainstream grid construction software includes HyperMesh, FEMB, Truegrid, ICEM, etc. [18]. The preprocessing required by LS-DYNA solver can be carried out by various means. Therefore, the preprocessing required by this numerical calculation is carried out by combining ANSYS/ICEM (ANSYS 14.5, Pittsburgh, PA, USA) and HyperMesh (HyperMesh 12.0, Altair, MI, USA). First, the hexahedral grid of the designed integral circumferential MEFP warhead is divided by ANSYS/ICEM, which will be divided. The mesh is imported into HyperMesh in the format of. K file to set the preprocessing of the finite-element model. To reduce the amount of calculation in the numerical simulation, according to the symmetry of the integral circumferential MEFP structure, the quarter finite-element model established is shown in Figure 3. The finite-element model shown in Figure 3 is divided into three parts: the main charge, the shell and the liner, and the air domain. Among them, the charge and the air adopt the Eulerian grid, and the shell and the liner adopt the Lagrangian grid, all of which are hexahedral solid elements with eight nodes. In addition, the multi-material Euler algorithm is used to simulate the forming process of integral circumferential MEFP warhead projectile.

### 3.2. Material Model

In the finite-element model of integral with shell and liner circumferential MEFP warhead as shown in Figure 3, 8701 explosive [19,20] is selected, high explosive material model and JWL (Jones Wilkins Lee) state equation are used, material parameters are shown in Table 2, and JWL state equation expression is shown in Equation (1).

General pressure expression of JWL equation of state [18]:

(1) $$p=A\left(1-\frac{\omega }{r_{1}V}\right)e^{-r_{1}V}+B\left(1-\frac{\omega }{r_{2}V}\right)e^{-r_{2}V}+\frac{\omega E}{V}$$

where A, B, $r_{1}$, $r_{2}$, and ω are material constants; V is the initial relative volume; E is the initial specific internal energy. ρ is the initial explosive density, $P_{CJ}$ is the detonation pressure and D is the detonation speed, p is the hydrostatic pressure.

The material of the shell and the liner is copper, the material model is JOHNSON_COOK, the state equation is described by GRUNEISEN, and the unit type is Lagrange grid. The material parameters are shown in Table 3, the expression of GRUNEISEN equation of state in compression state is shown in Equation (2), and the expression in expansion state is shown in Equation (3).

Expression of GRUNEISEN equation of state in compressed state [21,22]:

(2) $$p=\frac{\rho_{0}C^{2}\mu \left[1+\left(1-\frac{\gamma_{0}}{2}\right)\mu -\frac{\alpha }{2}\mu^{2}\right]}{{\left[1-\left(S_{1}-1\right)\mu -S_{2}\frac{\mu^{2}}{\mu +1}-S_{3}\frac{\mu^{3}}{{\left(\mu +1\right)}^{2}}\right]}^{2}}+\left(\gamma_{0}+\alpha \mu \right)E$$

Expression of GRUNEISEN equation of state in expansion state:

(3) $$\;p=\rho_{0}C^{2}\mu +\left(\gamma_{0}+\alpha \mu \right)E$$

where E is the initial internal energy, C is the intercept of the $v_{s}-v_{p}$ curve, $S_{1}$,$\;S_{2}$, and $S_{3}$ are the coefficients of the slope of the $v_{s}-v_{p}$ curve, $\gamma_{0}\;$is the GRUNEISEN coefficient, and α is the first-order volume, correction of $\gamma_{0}$.

MAT _NULL model is adopted for air materials. Air material parameters are shown in Table 4 [19]. The equation of state is described by linear polynomials, which is EOS_LINEAR_ POLYNOMIAL, and the element type is Euler grid.

## 4. Numerical Simulation Results of Integral Circumferential MEFP Warhead

### 4.1. Forming Results of Integral Circumferential MEFP Warhead

**Figure 4:**
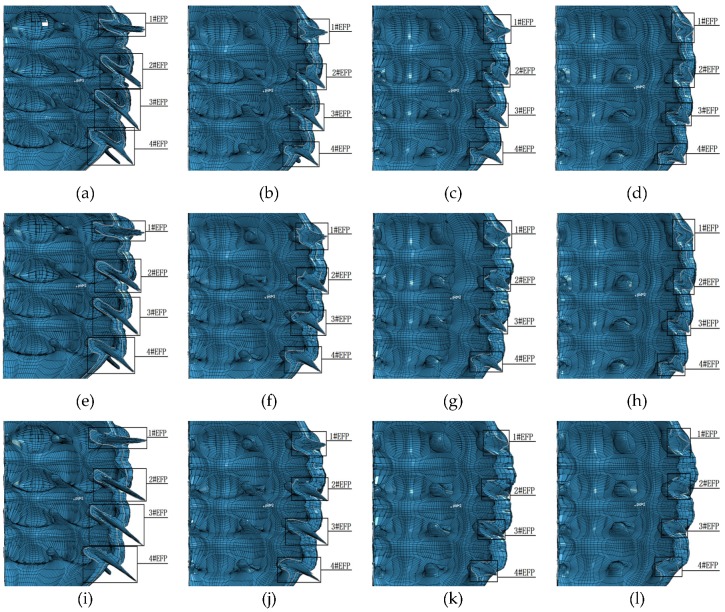
Forming results of integral circumferential MEFP warhead projectile: (**a**) Scheme 1 forming results; (**b**) Scheme 2 forming results; (**c**) Scheme 3 forming results; (**d**) Scheme 4 forming results; (**e**) Scheme 5 forming results; (**f**) Scheme 6 forming results; (**g**) Scheme 7 forming results; (**h**) Scheme 8 forming results; (**i**) Scheme 9 forming results; (**j**) Scheme 10 forming results; (**k**) Scheme 11 forming results; (**l**) Scheme 12 forming results.

The numerical simulation adopts single point initiation, and the unit is cm−μs−g. The forming results of the 12 structures shown in Table 1 are shown in Figure 4.

The structure of the integral circumferential MEFP is center symmetric, and the initiation point is in the straight line where the center of symmetry is located. Therefore, in the simulation results, the forming results of the 12 rows of liner are similar, so one of them is selected as the research object, as shown in Figure 4, where 1#EFP-4#EFP in (a–l) represents the forming results of one of the rows of liner. As can be seen from the molding results shown in Figure 4, when the curvature radius of inner wall and outer wall of the shell is constant, the shape of the formed projectile changes with the increase of the thickness at the center of the shell, as shown in Figure 4: a,e,i; b,f,j; c,g,k; As shown in (d), (h) and (l). When the wall thickness of the shaped shell is constant, the length diameter ratio of the formed projectile decreases gradually as the curvature radius of the inner and outer walls of the liner increases, as shown in Figure 4a–d; e–h; i–l. It can be seen from the forming results shown in Figure 4 that when the integrated design of the shell and the liner has variable wall thickness, the projectile forming shape of the integral circumferential MEFP warhead is not only related to the curvature radius of the inner and outer walls of the liner, but also affected by the change of the center thickness of the liner. That is to say, when the liner has variable wall thickness, the shape of the formed projectile is affected by the curvature radius of the inner wall, the curvature radius of the outer wall and the thickness of the center of the liner have a common effect. According to the forming effect of the integral circumferential MEFP warhead shown in Figure 4, when the curvature radius of the inner wall of the liner is between 10 mm and 12 mm, and the corresponding curvature radius of the outer wall of the liner is between 8 mm and 10 mm, the shape of the formed projectile is more reasonable.

When the initiation point is arranged at the center of the charge circle where the center point of the 1#EFP liner is as shown in Figure 3, due to the different positions of the detonation wave contacting the liner, the projectile will form a certain angle of dispersion during the forming process. Except for 1#EFP flying along the horizontal direction, the other formed projectiles shown in Figure 4 all have a certain flying angle. Therefore, when the fuse detonates the warhead in the near burst mode, the killing range of the target can be appropriately increased under the appropriate height of burst.

#### 1#EFP-4#EFP Forming Velocity of the Integral Circumferential MEFP Warhead

**Figure 5:**
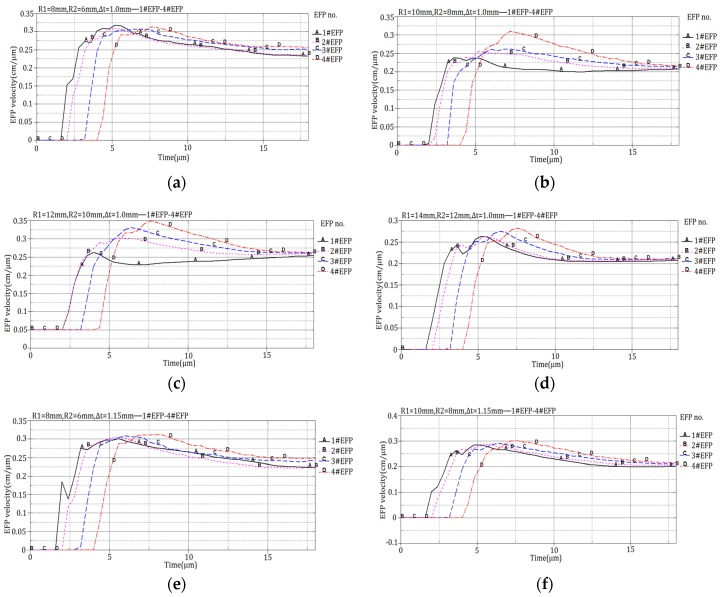

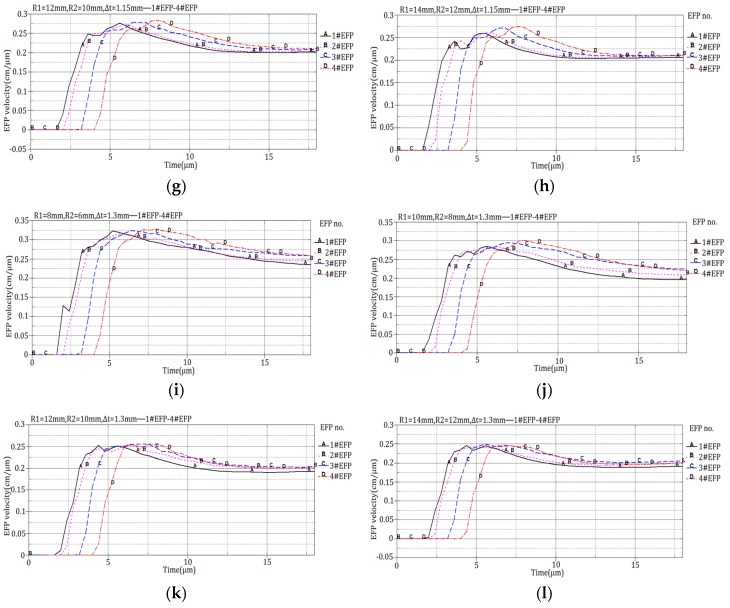
Projectile forming velocity of integral circumferential MEFP warhead: (**a**) Scheme 1 forming velocity; (**b**) Scheme 2 forming velocity; (**c**) Scheme 3 forming velocity; (**d**) Scheme 4 forming velocity; (**e**) Scheme 5 forming velocity; (**f**) Scheme 6 forming velocity; (**g**) Scheme 7 forming velocity; (**h**) Scheme 8 forming velocity; (**i**) Scheme 9 forming velocity; (j) Scheme 10 forming velocity; (**k**) Scheme 11 forming velocity; (**l**) Scheme 12 forming velocity.

The 12 design schemes shown in Table 1 and the corresponding velocity of 1#EFP -4#EFP shown in the forming results of each scheme in Figure 4 are shown in Figure 5.

It can be seen from Figure 5, When R1 = 8 mm, R2 = 6 mm, the projectile forming velocity reaches a stable state around 16 μs; when R1 = 10 mm, R2 = 8 mm, the projectile forming velocity reaches a stable state around 14 μs; when R1 = 12 mm, R2 = 10 mm, the projectile forming velocity reaches a stable state around 11 μs; when R1 = 14 mm, R2 = 12 mm, the projectile forming velocity reaches a stable state around 10.5 μs. The velocity of the formed projectile after it reaches stability is shown in Table 5.

**Figure 6:**
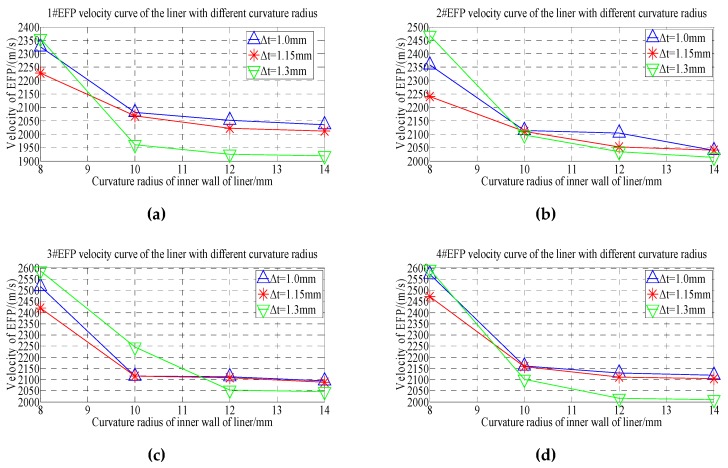
1#EFP-4#EFP velocity curve of the center of the liner with different thickness: (**a**) 1#EFP velocity curve; (**b**) 2#EFP velocity curve; (**c**) 3#EFP velocity curve;(**d**) 4#EFP velocity curve.

From the velocity of the forming projectile of 1#EFP-4#EFP shown in Table 5, it can be obtained that the velocity curve of the formed projectile of 1#EFP-4#EFP as shown in Figure 6.

As can be seen from the forming velocity curve of 1#EFP-4#EFP shown in Figure 6. With the increase of the radius of curvature, the velocity of formed projectile decreased. When the radius of curvature of the inner wall of the liner increases from 8 mm to 10 mm (the radius of curvature of the outer wall of the liner increases from 6 mm to 8 mm), the velocity of the formed projectile decreases greatly; when the radius of curvature of the inner wall of the liner increases from 10 mm to 14 mm (the radius of curvature of the outer wall of the liner increases from 8 mm to 12 mm), the velocity of the formed projectile decreases less.

**Figure 7:**
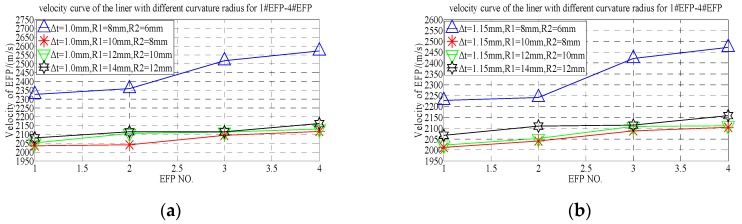

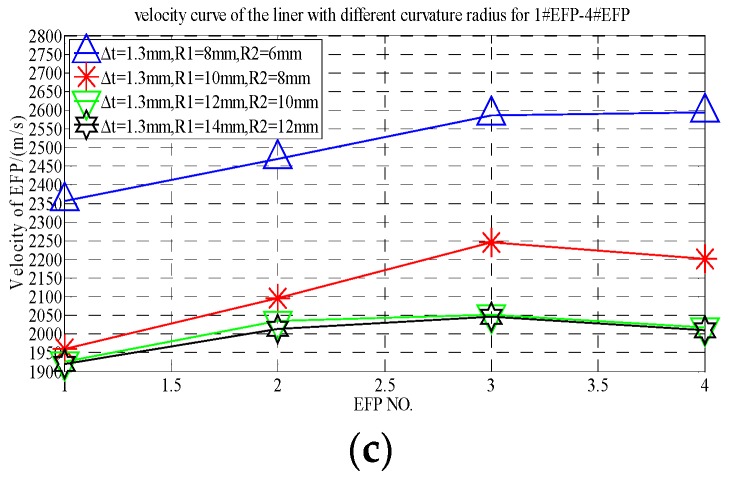
Velocity curve of the liner with different curvature radius for 1#EFP-4#EFP:(**a**) The center thickness of liner is 1 mm; (**b**) the center thickness of liner is 1.15 mm; (**c**) the center thickness of liner is 1.3 mm.

From the forming projectile velocity of 1#EFP-4#EFP shown in Table 5, the velocity curves of the center of the liner with thickness of 1.0 mm, 1.15 mm and 1.3 mm respectively are shown in Figure 7.

It can be seen from Figure 7 that when the thickness of the center of the liner is 1 mm–1.15 mm, the speed of 1#EFP-4#EFP increases gradually, as shown in Figure 7a,b; when the thickness at the center of the liner is 1.3 mm, the speed of the inner wall of the liner is 8 mm, the speed of 1#EFP-4#EFP increases gradually; when the curvature radius of the inner wall of the liner is 10–14 mm, the velocity of 1#EFP-3#EFP increase gradually, and the speed of 4#EFP starts to decrease, as shown in Figure 7c.

It can be seen from Figure 6 and Figure 7 that when the shell and the liner adopt the integrated design, the forming speed of EFP of the circumferential MEFP warhead with variable wall thickness of the liner is not only affected by the center thickness of the liner, but also by the curvature radius of the inner wall and the curvature radius of the outer wall of the liner.

It can be seen from the velocity curve of the formed projectile and the shaped of the formed projectile of each scheme that when the curvature radius of inner wall of the liner is 10–12 mm and the corresponding curvature radius of outer wall is 8–10 mm, the shape of formed projectile is better, and the velocity of 1#EFP-4#EFP is between 1900–2200m/s.

In the relevant literature [2,3,14,15,16,17] on the research of MEFP warhead, researchers studied the forming characteristics of MEFP from the aspects of the parameters (including the thickness, diameter, curvature radius), spacing, arrangement mode, and number of arrangement layers, etc. In the study of the influence of the parameters of the liner on the forming characteristics of the MEFP warhead, when the wall thickness of the liner is equal, the influence of the curvature radius, the diameter, and the thickness of the liner on the forming characteristics of the MEFP warhead is studied. To further study the influence of the curvature radius of the inner wall, the curvature radius of the outer wall and the thickness of the center of the liner on the MEFP warhead, when the liner has variable wall thickness, 24 schemes are redesigned based on the research of Part 2, Part 3 and Part 4, and numerical simulation is carried out.

## 5. Influence of the Parameters of the Liner on the Shape and Speed of MEFP

### 5.1. Structural Design of MEFP

According to the above simulation results, when R1 = 10–12 mm and R2 = 8–10 mm, the shape of the shaped projectile is better. To further study the influence of the curvature radius of the inner wall and outer wall and the different thickness at the center of liner on the shape and speed of the formed projectile with variable wall thickness, the parameters of the formed projectile are designed as shown in Table 6.

### 5.2. Simulation Results and Analysis

#### 5.2.1. Forming Results of R1 = 10 mm

**Figure 8:**
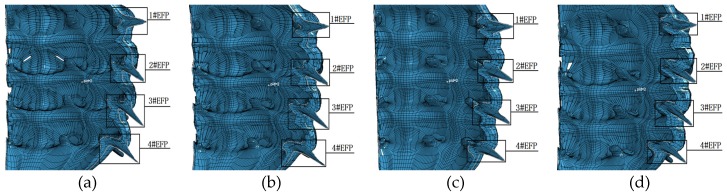

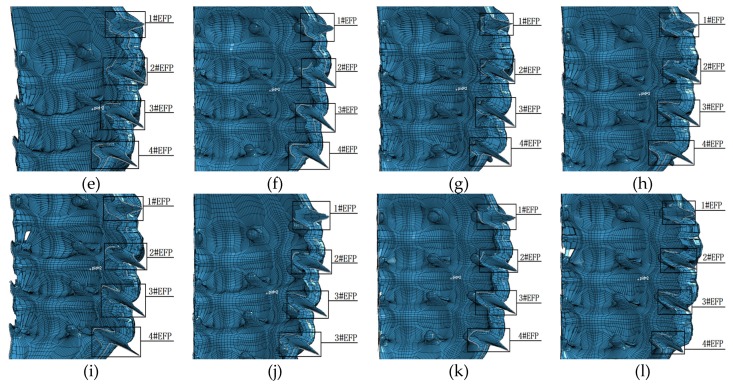
1#EFP-4#EFP formed shape of the liner which different curvature radius of outer wall and different center thickness:(**a**) R1 = 10 mm, R2 = 6 mm, Δt = 1.0 mm; (**b**) R1 = 10 mm, R2 = 7 mm, Δ t = 1.0 mm; (**c**) R1 = 10 mm, R2 = 9 mm, Δ t = 1.0 mm; (**d**) R1 = R2 = 10 mm, Δ t = 1.0 mm; (**e**) R1 = 10 mm, R2 = 6 mm, Δ t = 1.15 mm; (**f**) R1 = 10 mm, R2 = 7 mm, Δ t = 1.15 mm; (**g**) R1 = 10 mm, R2 = 9 mm, Δ t = 1.15 mm; (**h**) R1 = R2 = 10 mm, Δ t = 1.15 mm; (**i**) R1 = 10 mm, R2 = 6 mm, Δ t = 1.3 mm; (**j**) R1 = 10 mm, R2 = 7 mm, Δ t = 1.3 mm; (**k**) R1 = 10 mm, R2 = 9 mm, Δ t = 1.3 mm; (**l**) R1 = R2 = 10 mm, Δ t = 1.3 mm.

According to the structural parameters shown in Table 6, the integral circumferential MEFP is designed and simulated. When the radius of curvature of the inner wall of the liner is 10 mm and the thickness at the center of the liner is different, with the increase of the curvature radius of the outer wall of the liner, the forming results are shown in Figure 8.

**Figure 9:**
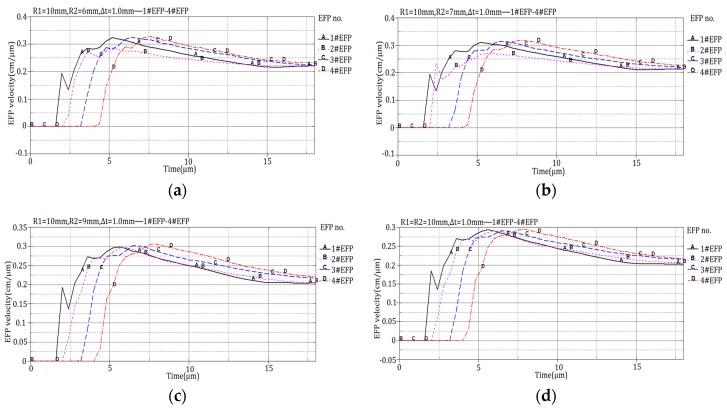

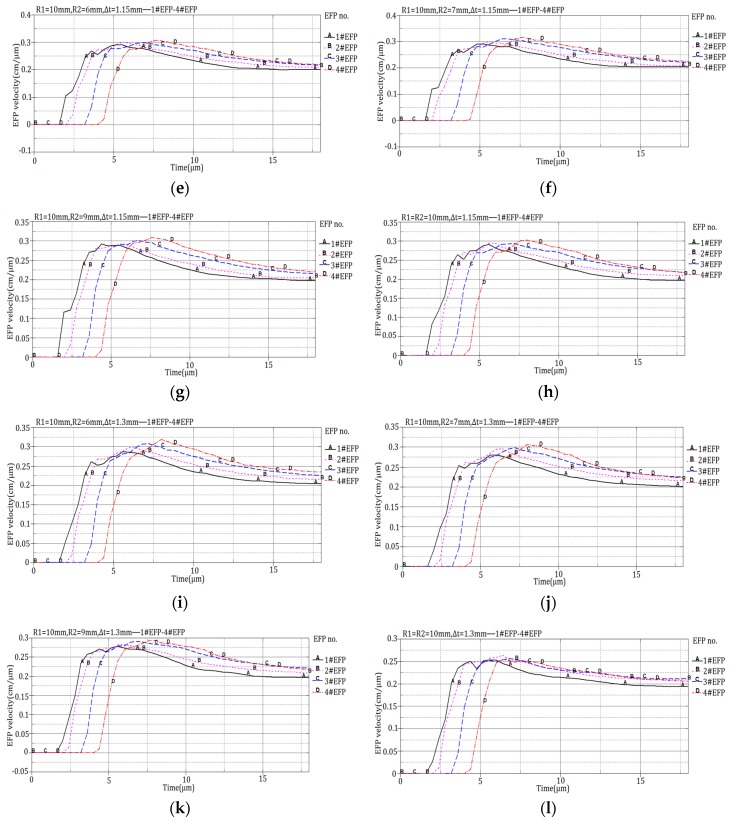
1#EFP-4#EFP formed velocity of the liner which different curvature radius of outer wall and different center thickness:(**a**) R1 = 10 mm, R2 = 6 mm, Δt = 1.0 mm; (**b**) R1 = 10 mm, R2 = 7 mm, Δ t = 1.0 mm; (**c**) R1 = 10 mm, R2 = 9 mm, Δ t = 1.0 mm; (**d**) R1 = R2 = 10 mm, Δ t = 1.0 mm; (**e**) R1 = 10 mm, R2 = 6 mm, Δ t = 1.15 mm; (**f**) R1 = 10 mm, R2 = 7 mm, Δ t = 1.15 mm; (**g**) R1 = 10 mm, R2 = 9 mm, Δ t = 1.15 mm; (**h**) R1 = R2 = 10 mm, Δ t = 1.15 mm; (**i**) R1 = 10 mm, R2 = 6 mm, Δ t = 1.3 mm; (**j**) R1 = 10 mm, R2 = 7 mm, Δ t = 1.3 mm; (**k**) R1 = 10 mm, R2 = 9 mm, Δ t = 1.3 mm; (**l**) R1 = R2 = 10 mm, Δ t = 1.3 mm.

It can be seen from Figure 8, when the thickness at the center of the liner is constant, the shape of the formed projectile does not change significantly with the increase of the curvature radius of the outer wall of the liner; when the curvature radius of the inner and outer walls of the liner is constant, the shape of the formed projectile changes and the ratio of length to diameter of the formed projectile decreases with the increase of the thickness at the center of the liner. It can be seen that when the curvature radius of the outer wall and the thickness of the center of the liner are increased, the shape of the formed projectile is greatly affected. The forming speed corresponding to the forming result shown in Figure 8 is shown in Figure 9.

According to the velocity curve of forming projectile shown in Figure 9, when the curvature radius of the inner wall of the liner is 10 mm, the forming velocity of 1#EFP-4#EFP is shown in Table 7.

**Figure 10:**
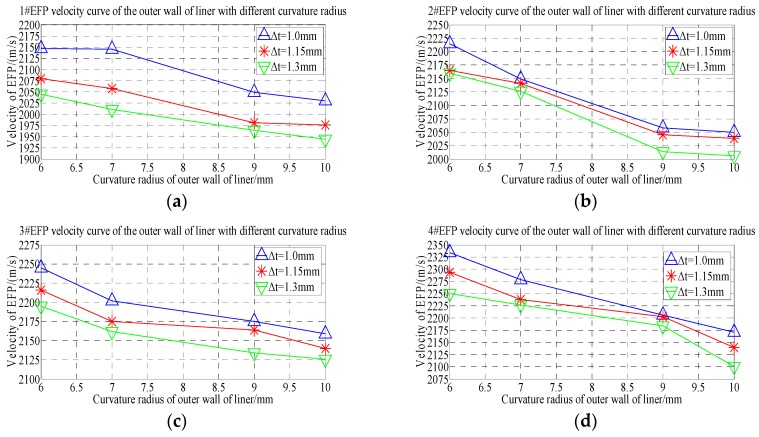
1#EFP-4#EFP velocity curve of the outer wall of the liner with different curvature radius: (**a**) 1#EFP velocity curve; (**b**) 2#EFP velocity curve; (**c**) 3#EFP velocity curve; (**d**) 4#EFP velocity curve.

According to the forming speed of 1#EFP-4#EFP shown in Table 7, the speed change rule curve is obtained that shown in Figure 10.

From the 1#EFP-4#EFP forming speed curve shown in Figure 10, it can be known that when the curvature radius of the inner wall of the liner is constant, with the increase of the curvature radius of the outer wall of the liner, the forming speed of 1#EFP-4#EFP decreased gradually. When the thickness of the center of the liner is 1.0 mm, the speed of 1#EFP decreases from 2147 m/s to 2030 m/s with the increase of the curvature radius of the outer wall of the liner, which decreased by about 5%; the speed of 2#EFP decreased from 2215 m/s to 2050 m/s, which decreased by about 7.5%; the speed of 3#EFP decreased from 2245 m/s to 2159 m/s, which decreased by about 4%; the speed of 4#EFP decreased from 2334 m/s to 2171 m/s, which decreased by about 7%. When the thickness of the center of the liner is 1.5 mm, the speed of 1#EFP decreases from 2080 m/s to 1976 m/s with the increase of the curvature radius of the outer wall of the liner, which decreased by about 5%; the speed of 2#EFP decreased from 2165 m/s to 2039 m/s, which decreased by about 6%; the speed of 3#EFP decreased from 2216 m/s to 2140 m/s, which decreased by about 3.5%; the speed of 4#EFP decreased from 2294 m/s to 2120 m/s, which decreased by about 7.6%. When the thickness of the center of the liner is 1.3 mm, the speed of 1#EFP decreases from 2045 m/s to 1944 m/s with the increase of the curvature radius of the outer wall of the liner, which decreased by about 5%; the speed of 2#EFP decreased from 2160 m/s to 2006 m/s, which decreased by about 7.1%; the speed of 3#EFP decreased from 2195 m/s to 2126 m/s, which decreased by about 3%; the speed of 4#EFP decreased from 2250 m/s to 2101 m/s, which decreased by about 6.6%.

**Figure 11:**
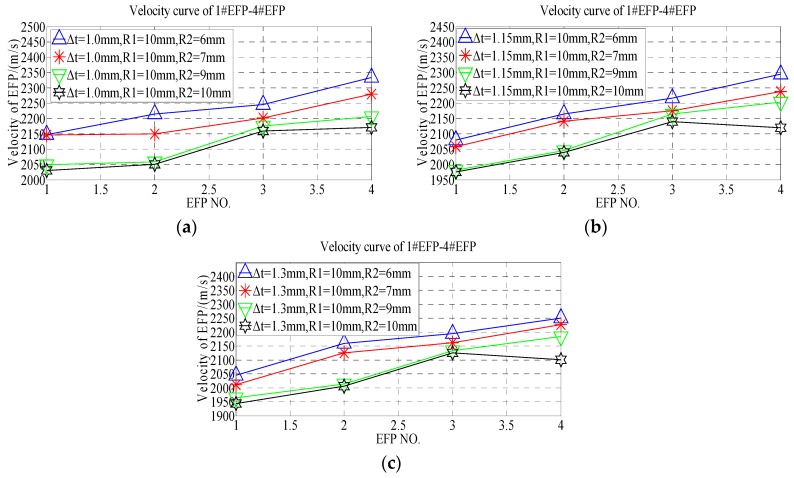
Velocity curve of 1#EFP-4#EFP: (**a**) the center thickness of liner is 1 mm; (**b**) the center thickness of liner is 1.15 mm; (**c**) the center thickness of liner is 1.3 mm.

According to the forming speed of 1#EFP-4#EFP shown in Table 7, the velocity change curve of #1EFP-4#EFP is obtained that shown in Figure 11.

It can be seen from Figure 11a that when ∆t = 1.0 mm, the forming speed of 1#EFP-4#EFP increases gradually and the increase slows down with the increase of the curvature radius of the outer wall of the liner. When R2 = 6 mm, the speed from 1#EFP to 4#EFP increased from 2147 m/s to 2334 m/s, which increased by about 8%; when R2 = 7 mm, the speed from 1#EFP to 4#EFP increased from 2146 m/s to 2279 m/s, which increased by about 5.8%; when R2 = 9 mm, the speed from 1#EFP to 4#EFP increased from 2049 m/s to 2206 m/s, which increased by about 7.1%; when R2 = 10 mm, the speed from 1#EFP to 4#EFP increased from 2030 m/s to 2171 m/s, which increased by about 6.5%. It can be seen from Figure 11b, when ∆t = 1.15 mm and the curvature radius of the outer wall of the liner is between 6–9 mm, the forming speed of 1#EFP-4#EFP increases gradually. When R2 = 6 mm, the speed from 1#EFP to 4#EFP increased from 2146 m/s to 2279 m/s, which increased by about 5.8%; when R2 = 7 mm, the speed from 1#EFP to 4#EFP increased from 2058 m/s to 2238 m/s, which increased by about 8%; when R2 = 9 mm, the speed from 1#EFP to 4#EFP increased from 1981 m/s to 2203 m/s, which increased by about 10%; when R2 = 10 mm, the speed from 1#EFP to 3#EFP increased from 1976 m/s to 2140 m/s, which increased by about 7.7%, and from 3#EFP to 4#EFP, decrease from 2140 m/s to 2120 m/s, which decreased by about 1%.

It can be seen from Figure 11c, when ∆t = 1.3 mm and R2 = 6 mm, the forming speed of 1#EFP-4#EFP increased gradually, the speed of 1#EFP-4#EFP increased from 2045 m/s to 2250 m/s, which increased by about 9.1%; when R2 = 6 mm, the forming speed of 1#EFP-4#EFP increased gradually, the speed of 1#EFP-4#EFP increased from 2011 m/s to 2227 m/s, which increased by about 9.7%; when R2 = 9 mm, the forming speed of 1#EFP-4#EFP increased gradually, the speed of 1#EFP-4#EFP increased from 1964 m/s to 2184 m/s, which increased by about 10.1%; when R2 = 10 mm, the forming speed of 1#EFP-3#EFP increased gradually, the speed of 1#EFP-3#EFP increased from 1964 m/s to 2184 m/s, which increased by about 8.6%, and the speed of from 3#EFP to 4#EFP, decrease from 2126 m/s to 2101 m/s, which decreased by about 1.2%.

**Figure 12:**
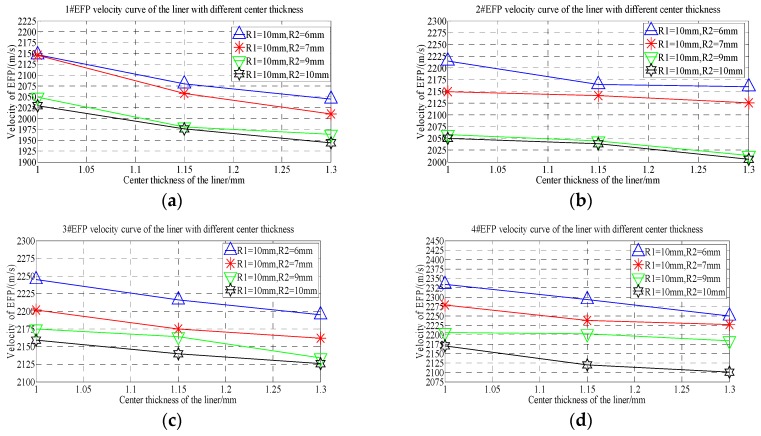
1#EFP-4#EFP velocity curve of the center of the liner with different thickness: (**a**) 1#EFP velocity curve; (**b**) 2#EFP velocity curve; (**c**) 3#EFP velocity curve; (**d**) 4#EFP velocity curve.

From the forming speed shown in Table 7, it can be seen that the forming speed curve of 1#EFP-4#EFP with the increase of thickness at the center of the liner, as shown in Figure 12.

It can be seen from Figure 12, When R2 = 6 mm, with the increase of the thickness at the center of the liner, the speed of 1#EFP decreased from 2147 m/s to 2045 m/s, which decreased by about 4.8%; the speed of 2#EFP decreased from 2215 m/s to 2160 m/s, which decreased by about 2.5%; the speed of 3#EFP decreased from 2245 m/s to 2195 m/s, which decreased by about 2.2%; the speed of 4#EFP decreased from 2334 m/s to 2250 m/s, which decreased by about 3.6%. When R2 = 7 mm, with the increase of the thickness at the center of the liner, the speed of 1#EFP decreased from 2146 m/s to 2011 m/s, which decreased by about 6.3%; the speed of 2#EFP decreased from 2149 m/s to 2126 m/s, which decreased by about 1.1%; the speed of 3#EFP decreased from 2202 m/s to 2162 m/s, which decreased by about 1.8%; the speed of 4#EFP decreased from 2279 m/s to 2238 m/s, which decreased by about 1.8%. When R2 = 9 mm, with the increase of the thickness at the center of the liner, the speed of 1#EFP decreased from 2049 m/s to 1964 m/s, which decreased by about 4.2%; the speed of 2#EFP decreased from 2058 m/s to 2014 m/s, which decreased by about 2.2%; the speed of 3#EFP decreased from 2175 m/s to 2134 m/s, which decreased by about 1.9%; the speed of 4#EFP decreased from 2206 m/s to 2184 m/s, which decreased by about 1%. When R2 = 10 mm, with the increase of the thickness at the center of the liner, the speed of 1#EFP decreased from 2030 m/s to 1944 m/s, which decreased by about 4.2%; the speed of 2#EFP decreased from 2050 m/s to 2006 m/s, which decreased by about 2.2%; the speed of 3#EFP decreased from 2159 m/s to 2126 m/s, which decreased by about 1.5%; the speed of 4#EFP decreased from 2171 m/s to 2101 m/s, which decreased by about 3.2%.

#### 5.2.2. Forming Results of R1 = 12 mm

**Figure 13:**
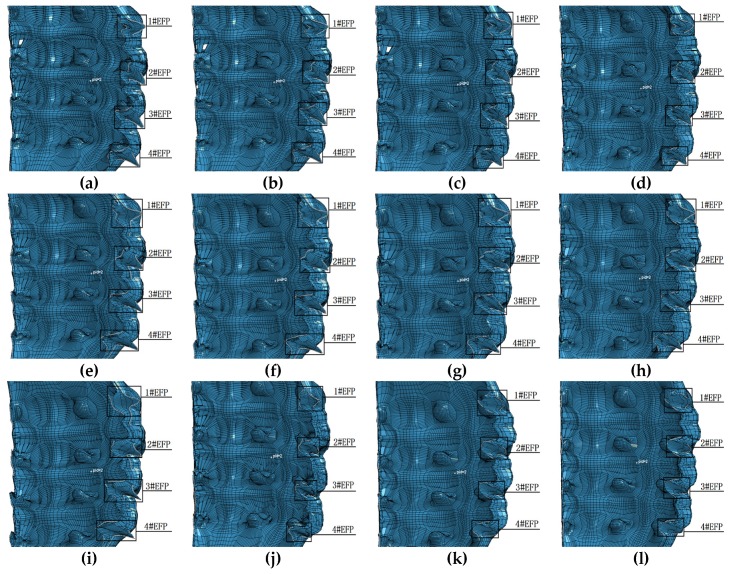
1#EFP-4#EFP formed shape of the liner which different curvature radius of outer wall and different center thickness:(**a**) R1 = 12 mm, R2 = 8 mm, Δt = 1.0 mm; (**b**) R1 = 12 mm, R2 = 9 mm, Δ t = 1.0 mm; (**c**) R1 = 12 mm, R2 = 11 mm, Δ t = 1.0 mm; (**d**) R1 = R2 = 12 mm, Δ t = 1.0 mm; (**e**) R1 = 12 mm, R2 = 8 mm, Δ t = 1.15 mm; (**f**) R1 = 12 mm, R2 = 9 mm, Δ t = 1.15 mm; (**g**) R1 = 12 mm, R2 = 11 mm, Δ t = 1.15 mm; (**h**) R1 = R2 = 12 mm, Δ t = 1.15 mm; (**i**) R1 = 12 mm, R2 = 8 mm, Δ t = 1.3 mm; (**j**) R1 = 12 mm, R2 = 9 mm, Δ t = 1.3 mm; (**k**) R1 = 12 mm, R2 = 11 mm, Δ t = 1.3 mm; (**l**) R1 = R2 = 12 mm, Δ t = 1.3 mm.

According to the structural parameters shown in Table 6, the integral circumferential MEFP is designed and simulated. When the radius of curvature of the inner wall of the liner is 12 mm and the thickness at the center of the liner is different, with the increase of the curvature radius of the outer wall of the liner, the forming results are shown in Figure 13.

It can be seen from Figure 13, when R1 = 12 mm, Δt = 1.0 mm, with the increase of the curvature radius of the outer wall of the liner, the shape of the formed projectile has no obvious change; with the increase of the thickness of the center of the liner and the curvature radius of the outer wall of the liner, the shape of the formed projectile changes. When R1 = 12 mm, Δt = 1.3 mm, with the increase of the curvature radius of the outer wall of the liner, the shape of the formed projectile is approximately spherical. According to the forming results of different thickness at the center of the liner and different curvature radius of the outer wall when R1 = 10 mm as shown in Figure 9, With the increase of the curvature radius and the thickness of the center of the liner at the same time, the influence on the formed projectile is greater.

**Figure 14:**
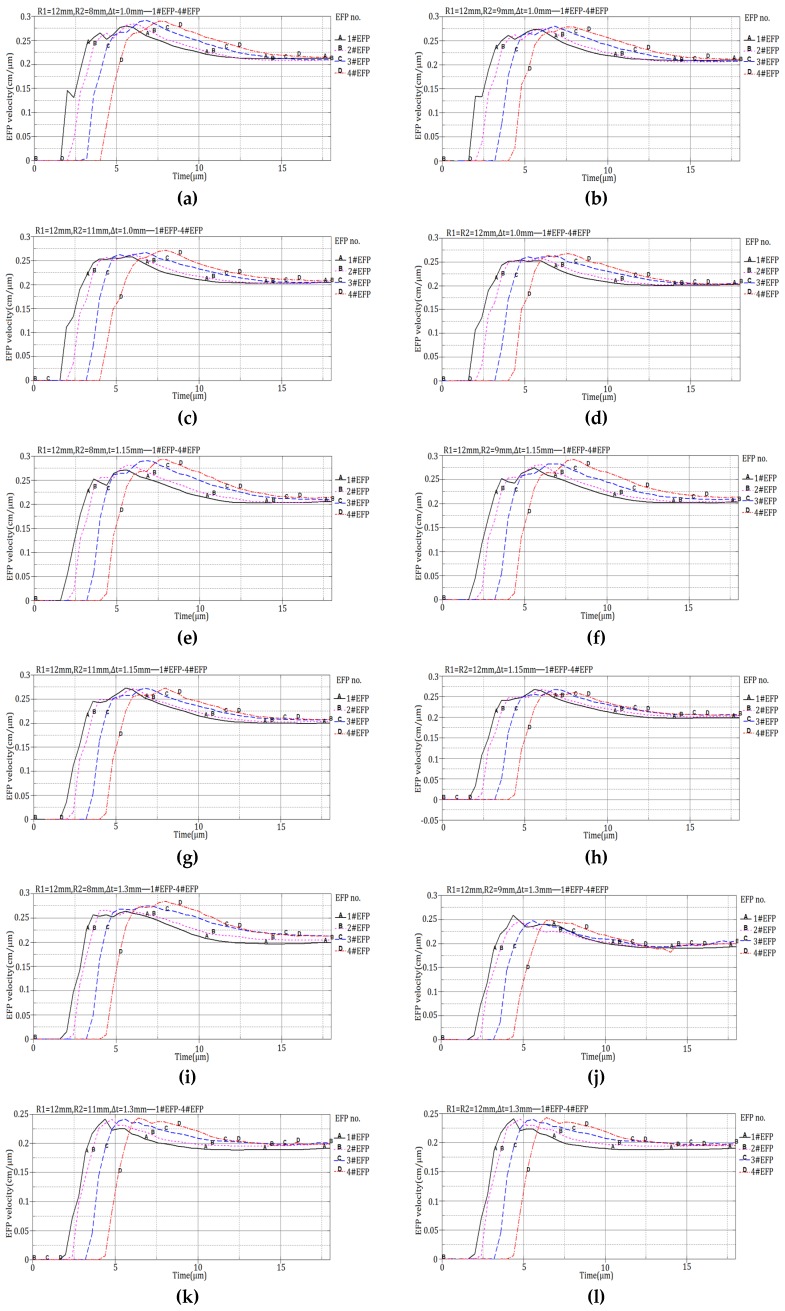
1#EFP-4#EFP formed velocity of the liner which different curvature radius of outer wall and different center thickness:(**a**) R1 = 12 mm, R2 = 8 mm, Δt = 1.0 mm; (**b**) R1 = 12 mm, R2 = 9 mm, Δ t = 1.0 mm; (**c**) R1 = 12 mm, R2 = 11 mm, Δ t = 1.0 mm; (**d**) R1 = R2 = 12 mm, Δ t = 1.0 mm; (**e**) R1 = 12 mm, R2 = 8 mm, Δ t = 1.15 mm; (f) R1 = 12 mm, R2 = 9 mm, Δ t = 1.15 mm; (**g**) R1 = 12 mm, R2 = 11 mm, Δ t = 1.15 mm; (**h**) R1 = R2 = 12 mm, Δ t = 1.15 mm; (**i**) R1 = 12 mm, R2 = 8 mm, Δ t = 1.3 mm; (**j**) R1 = 12 mm, R2 = 9 mm, Δ t = 1.3 mm; (**k**) R1 = 12 mm, R2 = 11 mm, Δ t = 1.3 mm; (**l**) R1 = R2 = 12 mm, Δ t = 1.3 mm.

The forming speed corresponding to the forming result shown in Figure 13 is shown in Figure 14.

According to the velocity curve of forming projectile shown in Figure 14, when the curvature radius of the inner wall of the liner is 12 mm, the forming velocity of 1#EFP-4#EFP is shown in Table 8.

**Figure 15:**
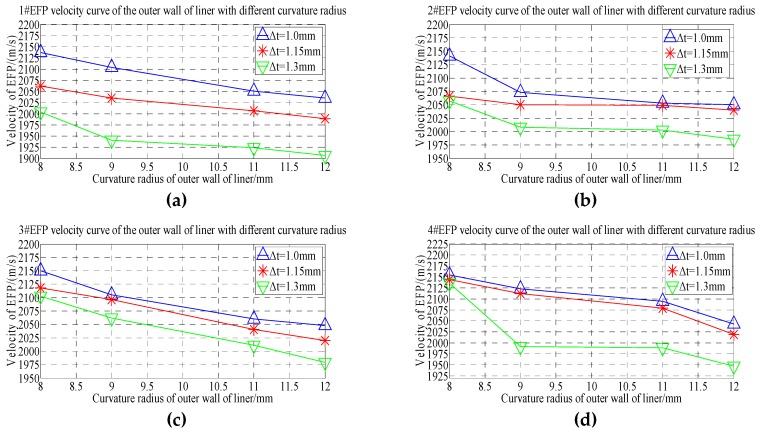
1#EFP-4#EFP velocity curve of the center of the liner with different thickness: (**a**) 1#EFP velocity curve; (**b**) 2#EFP velocity curve; (**c**) 3#EFP velocity curve;(**d**) 4#EFP velocity curve.

According to the forming speed of 1#EFP-4#EFP shown in Table 8, the speed change rule curve is obtained that shown in Figure 15.

From the 1#EFP-4#EFP forming speed curve shown in Figure 15, it can be known that when the curvature radius of the inner wall of the liner is constant, with the increase of the curvature radius of the outer wall of the liner, the forming speed of 1#EFP-4#EFP decreased gradually. When the thickness of the center of the liner is 1.0 mm, the speed of 1#EFP decreases from 2137 m/s to 2035 m/s with the increase of the curvature radius of the outer wall of the liner, which decreased by about 4.8%; the speed of 2#EFP decreased from 2141 m/s to 2050 m/s, which decreased by about 4.3%; the speed of 3#EFP decreased from 2150 m/s to 2055 m/s, which decreased by about 4.4%; the speed of 4#EFP decreased from 2155 m/s to 2043 m/s, which decreased by about 5.2%. When the thickness of the center of the liner is 1.15 mm, the speed of 1#EFP decreases from 2062 m/s to 1989 m/s with the increase of the curvature radius of the outer wall of the liner, which decreased by about 3.5%; the speed of 2#EFP decreased from 2066 m/s to 2040 m/s, which decreased by about 1.3%; the speed of 3#EFP decreased from 2118 m/s to 2020 m/s, which decreased by about 4.6%; the speed of 4#EFP decreased from 2144 m/s to 2019 m/s, which decreased by about 5.8%. When the thickness of the center of the liner is 1.3 mm, the speed of 1#EFP decreases from 2004 m/s to 1907 m/s with the increase of the curvature radius of the outer wall of the liner, which decreased by about 4.8%; the speed of 2#EFP decreased from 2057 m/s to 1986 m/s, which decreased by about 3.5%; the speed of 3#EFP decreased from 2103 m/s to 1979 m/s, which decreased by about 5.9%; the speed of 4#EFP decreased from 2136 m/s to 1947 m/s, which decreased by about 8.8%.

**Figure 16:**
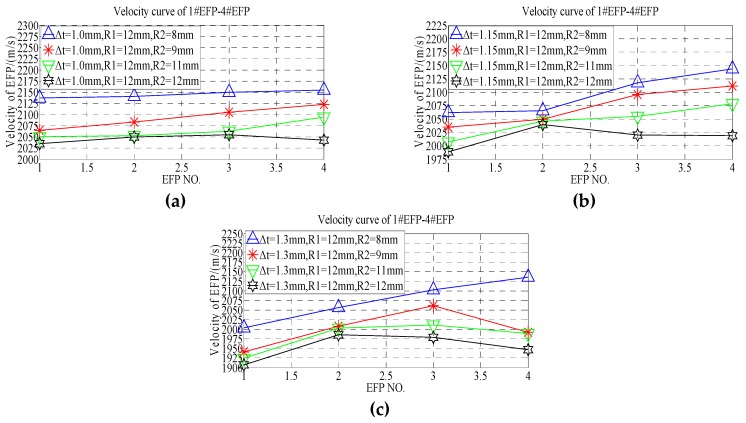
Velocity curve of 1#EFP-4#EFP:(**a**) the center thickness of liner is 1 mm; (**b**) the center thickness of liner is 1.15 mm; (**c**) the center thickness of liner is 1.3 mm.

According to the forming speed of 1#EFP-4#EFP shown in Table 8, the velocity change curve of # 1EFP-4EFP is obtained that shown in Figure 16.

According to the velocity comparison curve of 1#EFP-4#EFP shown in Figure 16a, when the thickness of the center of the liner is 1.0 mm, the curvature radius of the liner outer wall is between 8 mm and 11 mm, the velocity of 1#EFP-4#EFP increases gradually. However, the velocity of 1#EFP-3#EFP increases gradually, when the curvature radius of the liner outer wall is 12 mm, while the velocity of 4#EFP decreases. In Figure 16a, when R2 = 8 mm, the speed from 1#EFP to 4#EFP increases from 2137 m/s to 2155 m/s, which increased by about 1%; when R2 = 9 mm, the speed from 1#EFP to 4#EFP increases from 2065 m/s to 2123 m/s, which increased by about 2.7%; when R2 = 11 mm, the speed from 1#EFP to 4#EFP increases from 2051 m/s to 2095 m/s, which increased by about 2.1%; when R2 = 12 mm, the speed from 1#EFP to 4#EFP increases from 2035 m/s to 2043 m/s, which increased by about 0.4%. According to the velocity comparison curve of 1#EFP-4#EFP shown in Figure 16b, when the thickness of the center of the liner is 1.15 mm, the curvature radius of the liner outer wall is between 8 mm and 11 mm, the velocity of 1#EFP-4#EFP increases gradually. However, the velocity of 1#EFP-2#EFP increases gradually, when the curvature radius of the liner outer wall is 12 mm, while the velocity of 2#EFP-4#EFP decreases gradually. In Figure 16b, when R2 = 8 mm, the speed from 1#EFP to 4#EFP increases from 2062 m/s to 2144 m/s, which increased by about 3.8%; when R2 = 9 mm, the speed from 1#EFP to 4#EFP increases from 2035 m/s to 2112 m/s, which increased by about 3.6%; when R2 = 11 mm, the speed from 1#EFP to 4#EFP increases from 2007 m/s to 2079 m/s, which increased by about 3.5%; when R2 = 12 mm, the speed from 1#EFP to 2#EFP increases from 1989 m/s to 2040 m/s, which increased by about 2.5%; the speed from 2#EFP to 4#EFP increases from 2040 m/s to 2019 m/s, which decreased by about 1%. According to the velocity comparison curve of 1#EFP-4#EFP shown in Figure 16c, when the thickness of the center of the liner is 1.3 mm, the curvature radius of the liner outer wall is 8 mm, the velocity of 1#EFP-4#EFP increases gradually. However, the velocity of 1#EFP-3#EFP increases gradually, when the curvature radius of the liner outer wall is between 9 mm and 11 mm, while the velocity of 3#EFP-4#EFP decreases gradually; the velocity of 1#EFP-2#EFP increases gradually, while the velocity of 2#EFP-4#EFP decreases gradually, when the curvature radius of the liner outer wall is 12 mm. In Figure 16c, when R2 = 8 mm, the speed from 1#EFP to 4#EFP increases from 2004 m/s to 2136 m/s, which increased by about 6.2%. When R2 = 9 mm, the speed from 1#EFP to 3#EFP increases from 1941 m/s to 2062 m/s, which increased by about 6.1%, while the speed from 3#EFP to 4#EFP decreases from 2062 m/s to 1992m/s, which decreased by about 3.4%. When R2 = 11 mm, the speed from 1#EFP to 2#EFP increases from 1924 m/s to 2003 m/s, which increased by about 3.9%, while the speed from 2#EFP to 4#EFP decreases from 2003 m/s to 1989 m/s, which decreased by about 0.7%. When R2 = 12 mm, the speed from 1#EFP to 2#EFP increases from 1907 m/s to 1986 m/s, which increased by about 4%, while the speed from 2#EFP to 4#EFP decreases from 1986 m/s to 1947 m/s, which decreased by about 2%.

**Figure 17:**
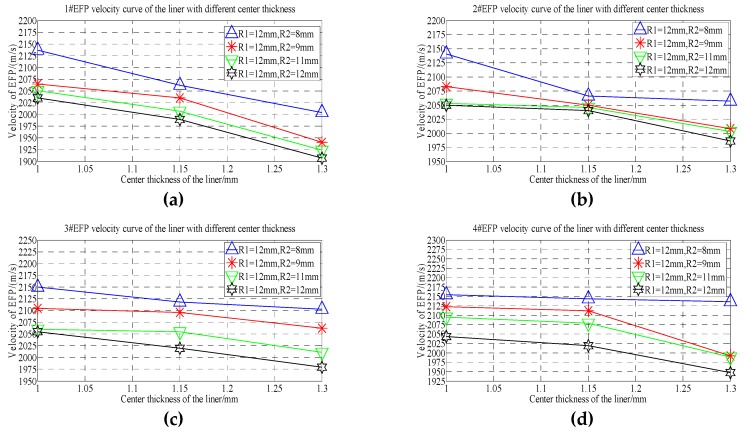
1#EFP-4#EFP velocity curve of the center of the liner with different thickness: (**a**) 1#EFP velocity curve; (**b**) 2#EFP velocity curve; (**c**) 3#EFP velocity curve; (**d**) 4#EFP velocity curve.

From the forming speed shown in Table 8, it can be seen that the forming speed curve of 1#EFP-4#EFP with the increase of thickness at the center of the liner, as shown in Figure 17.

As can be seen from Figure 17, when R2 = 8 mm, with the increase of the thickness at the center of the liner, the speed of 1#EFP decreased from 2137 m/s to 2004 m/s, which decreased by about 6.2%; the speed of 2#EFP decreased from 2141 m/s to 2057 m/s, which decreased by about 3.9%; the speed of 3#EFP decreased from 2150 m/s to 2103 m/s, which decreased by about 2.2%; the speed of 4#EFP decreased from 2155 m/s to 2136 m/s, which decreased by about 0.9%. When R2 = 9 mm, with the increase of the thickness at the center of the liner, the speed of 1#EFP decreased from 2065 m/s to 1941 m/s, which decreased by about 6%; the speed of 2#EFP decreased from 2083 m/s to 2008 m/s, which decreased by about 3.6%; the speed of 3#EFP decreased from 2105 m/s to 2062 m/s, which decreased by about 2.1%; the speed of 4#EFP decreased from 2123 m/s to 1992 m/s, which decreased by about 6.2%. When R2 = 11 mm, with the increase of the thickness at the center of the liner, the speed of 1#EFP decreased from 2051 m/s to 1924 m/s, which decreased by about 6.2%; the speed of 2#EFP decreased from 2053 m/s to 2003 m/s, which decreased by about 2.4%; the speed of 3#EFP decreased from 2060 m/s to 2011 m/s, which decreased by about 2.4%; the speed of 4#EFP decreased from 2095 m/s to 1989 m/s, which decreased by about 5.1%. When R2 = 12 mm, with the increase of the thickness at the center of the liner, the speed of 1#EFP decreased from 2035 m/s to 1907 m/s, which decreased by about 6.3%; the speed of 2#EFP decreased from 2050 m/s to 1986 m/s, which decreased by about 3.1%; the speed of 3#EFP decreased from 2055 m/s to 1979 m/s, which decreased by about 3.7%; the speed of 4#EFP decreased from 2043 m/s to 1947 m/s, which decreased by about 4.7%.

## 6. Conclusions

In this study, ANSYS/ICEM-HyperMesh is used to mesh and set the boundary conditions of the small-caliber circumferential MEFP ammunition structure, and LS-DYNA is used to solve the problem. First, the numerical simulation of 12 kinds of circumferential MEFP ammunition structures (as shown in Table 1) is carried out. Through the numerical simulation, it was found that when the curvature radius of the inner wall of the liner was 10 mm and 12 mm, and the corresponding curvature radius of the outer wall was 8 mm and 10 mm, the shape of the formed projectile was better and the velocity of the formed projectile ranged from 1900–2200 m/s. On this basis, the influence of the curvature radius of the outer wall of the liner and the thickness of the center of the liner on the shape and velocity of the formed projectile was further studied, and the following conclusions were drawn:

(1) It is feasible to use the method of combining ANSYS/ICEM-HyperMesh to divide the finite-element mesh. The finite-element mesh with high quality can be obtained, and set the boundary conditions required by LS-DYNA solution, to obtain satisfactory numerical simulation results;

(2) When the liner has variable wall thickness, the shape of the formed projectile is affected by the curvature radius of the inner wall of the liner, the curvature radius of the outer wall of the liner, and the thickness at the center of the liner. When the curvature radius of the inner wall and the corresponding outer wall curvature radius of the liner increase at the same time, the shape of the projectile is greatly affected, and the speed of the projectile decreases as the curvature radius of the inner and outer walls of the liner increases;

(3) When the thickness of the center of the liner and the curvature radius of the inner wall of the liner are constant, the velocity of forming projectile decreases with the increase of the curvature radius of the outer wall of the liner. When R1 = 10 mm, the speed of the 1#EFP-4#EFP decreased by between 3% and 7.5%; when R1 = 12 mm, the speed of the 1#EFP-4#EFP decreased by between 1% and 9%;

(4) When the curvature radius of the outer wall and the inner wall of the liner are constant, the velocity of the 1#EFP-4#EFP increases first and then decreases with the increase of the thickness of the liner center and the curvature radius of the liner outer wall. Therefore, with the increase of the thickness of the center of the liner and the curvature radius of the liner outer wall, the velocity of the 1#EFP-4#EFP is more affected;

(5) When the curvature radius of the outer wall and inner wall of the liner are constant, the velocity of 1#EFP-4#EFP decreases with the increase of the thickness of the liner center, and the decrease range is between 1% and 6.5%. According to conclusion (3), the influence of the increase of the curvature radius of the outer wall of the liner on the velocity of formed projectile is slightly greater than that of the increase of the thickness at the center of the liner;

(6) When the curvature radius of the outer wall and the thickness of the center of the liner are increased, the shape of the formed projectile is more affected by the thickness of the center of the liner than by the curvature radius of the outer wall.

## Figures and Tables

**Table 1:** Structural parameter design of integral circumferential MEFP.

Basic Parameters of MEFP	Scheme	Center Thickness of Liner (Δt)	Curvature Radius of Inner Wall of Liner (R1)	Curvature Radius of Outer Wall of Liner (R2)
The charging height is 52 mm, the diameter of the charging is 37 mm, the shell thickness is 1.5 mm, and the diameter of the liner is 9 mm	1	1 mm	8 mm	6 mm
	2		10 mm	8 mm
	3		12 mm	10 mm
	4		14 mm	12 mm
	5	1.15 mm	8 mm	6 mm
	6		10 mm	8 mm
	7		12 mm	10 mm
	8		14 mm	12 mm
	9	1.3 mm	8 mm	6 mm
	10		10 mm	8 mm
	11		12 mm	10 mm
	12		14 mm	12 mm

**Table 2:** Material parameters for 8701 explosive.

Material	ρ (g/cm^3^)	D (cm/μs)	$P_{CJ}\;(\mathrm{GPa})$	E (GPa)	A (GPa)	B (GPa)	$r_{1}$	$r_{2}$	ω	$v_{0}$
Explosive	1.71	0.83	28.6	8.5	524.23	7.678	34	1.1	0.34	1

**Table 3:** Material parameters for Copper.

Material	ρ (g/cm^3^)	G (GPa)	A (MPa)	B (MPa)	n	C	m	$T_{m}\;(K)$	$T_{room}\;(K)$	Γ	$c_{0}$ (cm/μs)	S
Copper	8.93	46.5	90	292	0.31	0.025	1.09	1356	293	2.02	0.39	1.49

**Table 4:** Material parameters of air.

Material	ρ $\left(g/cm^{3}\right)$	γ	$C_{p}$ (kJ/kg·k)	$C_{p}$ (kJ/kg·k)	T(K)	$E_{0}$ $\left(\mathrm{kJ}/kg^{-1}\right)$
Air	1.225	1.4	1.005	0.718	288.2	206,800

**Table 5:** 1#EFP-4#EFP formed velocity.

Projectile Number	Radius of Curvature	Δt = 1 mm	Δt = 1.15 mm	Δ = 1.3 mm
1#EFP	R1 = 8 mm, R2 = 6 mm	2326 m/s	2228 m/s	2357 m/s
	R1 = 10 mm, R2 = 8 mm	2081 m/s	2068 m/s	1961 m/s
	R1 = 12 mm, R2 = 10 mm	2052 m/s	2022 m/s	1926 m/s
	R1 = 14 mm, R2 = 12 mm	2035 m/s	2012 m/s	1920 m/s
2#EFP	R1 = 8 mm, R2 = 6 mm	2359 m/s	2241 m/s	2469 m/s
	R1 = 10 mm, R2 = 8 mm	2114 m/s	2110 m/s	2097 m/s
	R1 = 12 mm, R2 = 10 mm	2105 m/s	2053 m/s	2035 m/s
	R1 = 14 mm, R2 = 12 mm	2040 m/s	2041 m/s	2014 m/s
3#EFP	R1 = 8 mm, R2 = 6 mm	2517 m/s	2421 m/s	2586 m/s
	R1 = 10 mm, R2 = 8 mm	2115 m/s	2115 m/s	2246 m/s
	R1 = 12 mm, R2 = 10 mm	2113 m/s	2108 m/s	2053 m/s
	R1 = 14 mm, R2 = 12 mm	2094 m/s	2088 m/s	2046 m/s
4#EFP	R1 = 8 mm, R2 = 6 mm	2573 m/s	2472 m/s	2594 m/s
	R1 = 10 mm, R2 = 8 mm	2161 m/s	2158 m/s	2102 m/s
	R1 = 12 mm, R2 = 10 mm	2130 m/s	2111 m/s	2017 m/s
	R1 = 14 mm, R2 = 12 mm	2120 m/s	2104 m/s	2011 m/s

**Table 6:** Structural design of MEFP.

Basic Parameters of MEFP	Center Thickness of Liner (Δt)	Curvature Radius of Inner Wall of Liner (R1)	Curvature Radius of Outer Wall of Liner (R2)
The charging height is 52 mm, the diameter of the charging is 37 mm, the shell thickness is 1.5 mm, and the diameter of the liner is 9 mm	1 mm	10 mm	6 mm
			7 mm
			9 mm
			10 mm
		12 mm	8 mm
			9 mm
			11 mm
			12 mm
	1.15 mm	10 mm	6 mm
			7 mm
			9 mm
			10 mm
		12 mm	8 mm
			9 mm
			11 mm
			12 mm
	1.3 mm	10 mm	6 mm
			7 mm
			9 mm
			10 mm
		12 mm	8 mm
			9 mm
			11 mm
			12 mm

**Table 7:** 1#EFP-4#EFP formed velocity.

Projectile Number	Curvature Radius	Δt = 1 mm	Δt = 1.15 mm	Δt = 1.3 mm
1#EFP	R1 = 10 mm, R2 = 6 mm	2147 m/s	2080 m/s	2045 m/s
	R1 = 10 mm, R2 = 7 mm	2146 m/s	2058 m/s	2011 m/s
	R1 = 10 mm, R2 = 9 mm	2049 m/s	1981 m/s	1964 m/s
	R1 = 10 mm, R2 = 10 mm	2030 m/s	1976 m/s	1944 m/s
2#EFP	R1 = 10 mm, R2 = 6 mm	2215 m/s	2165 m/s	2160 m/s
	R1 = 10 mm, R2 = 7 mm	2149 m/s	2141 m/s	2126 m/s
	R1 = 10 mm, R2 = 9 mm	2058 m/s	2045 m/s	2014 m/s
	R1 = 10 mm, R2 = 10 mm	2050 m/s	2039 m/s	2006 m/s
3#EFP	R1 = 10 mm, R2 = 6 mm	2245 m/s	2216 m/s	2195 m/s
	R1 = 10 mm, R2 = 7 mm	2202 m/s	2175 m/s	2162 m/s
	R1 = 10 mm, R2 = 9 mm	2175 m/s	2164 m/s	2134 m/s
	R1 = 10 mm, R2 = 10 mm	2159 m/s	2140 m/s	2126 m/s
4#EFP	R1 = 10 mm, R2 = 6 mm	2334 m/s	2294 m/s	2250 m/s
	R1 = 10 mm, R2 = 7 mm	2279 m/s	2238 m/s	2227 m/s
	R1 = 10 mm, R2 = 9 mm	2206 m/s	2203 m/s	2184 m/s
	R1 = 10 mm, R2 = 10 mm	2171 m/s	2120 m/s	2101 m/s

**Table 8:** 1#EFP-4#EFP formed velocity for R1 = 12 mm.

Projectile Number	Radius of Curvature	Δt = 1 mm	Δt = 1.15 mm	Δt = 1.3 mm
1#EFP	R1 = 12 mm, R2 = 8 mm	2137 m/s	2062 m/s	2004 m/s
	R1 = 12 mm, R2 = 9 mm	2065 m/s	2035 m/s	1941 m/s
	R1 = 12 mm, R2 = 11 mm	2051 m/s	2007 m/s	1924 m/s
	R1 = 12 mm, R2 = 12 mm	2035 m/s	1989 m/s	1907 m/s
2#EFP	R1 = 12 mm, R2 = 8 mm	2141 m/s	2066 m/s	2057 m/s
	R1 = 12 mm, R2 = 9 mm	2083 m/s	2050 m/s	2008 m/s
	R1 = 12 mm, R2 = 11 mm	2053 m/s	2046 m/s	2003 m/s
	R1 = 12 mm, R2 = 12 mm	2050 m/s	2040 m/s	1986 m/s
3#EFP	R1 = 12 mm, R2 = 8 mm	2150 m/s	2118 m/s	2103 m/s
	R1 = 12 mm, R2 = 9 mm	2105 m/s	2096 m/s	2062 m/s
	R1 = 12 mm, R2 = 11 mm	2060 m/s	2055 m/s	2011 m/s
	R1 = 12 mm, R2 = 12 mm	2055 m/s	2020 m/s	1979 m/s
4#EFP	R1 = 12 mm, R2 = 8 mm	2155 m/s	2144 m/s	2136 m/s
	R1 = 12 mm, R2 = 9 mm	2123 m/s	2112 m/s	1992 m/s
	R1 = 12 mm, R2 = 11 mm	2095 m/s	2079 m/s	1989 m/s
	R1 = 12 mm, R2 = 12 mm	2043 m/s	2019 m/s	1947 m/s
